# Evaluation of Sleep Practices and Knowledge in Neonatal Healthcare

**DOI:** 10.1097/ANC.0000000000001102

**Published:** 2023-08-14

**Authors:** Eline R. de Groot, Mary-Anne Ryan, Chanel Sam, Olaf Verschuren, Thomas Alderliesten, Jeroen Dudink, Agnes van den Hoogen

**Affiliations:** Department of Neonatology, Wilhelmina Children's Hospital (Mss de Groot and Sam and Drs Alderliesten, Dudink, and van den Hoogen), and Brain Centre Rudolf Magnus (Drs Alderliesten and Dudink), University Medical Center Utrecht, Utrecht, the Netherlands; INFANT Centre, University College Cork, Cork, Ireland (Ms Ryan); Department of Neonatology, Cork University Maternity Hospital, Cork, Ireland (Ms Ryan); and UMC Utrecht Brain Center and Center of Excellence for Rehabilitation Medicine (Dr Verschuren), Utrecht University (Dr van den Hoogen), Utrecht, the Netherlands.

**Keywords:** developmental neonatal care, neonate, sleep, sleep knowledge, sleep practice

## Abstract

**Background:**

Developmental care is designed to optimize early brain maturation by integrating procedures that support a healing environment. Protecting preterm sleep is important in developmental care. However, it is unclear to what extent healthcare professionals are aware of the importance of sleep and how sleep is currently implemented in the day-to-day care in the neonatal intensive care unit (NICU).

**Purpose:**

Identifying the current state of knowledge among healthcare professionals regarding neonatal sleep and how this is transferred to practice.

**Methods:**

A survey was distributed among Dutch healthcare professionals. Three categories of data were sought, including (1) demographics of respondents; (2) questions relating to sleep practices; and (3) objective knowledge questions relating to sleep physiology and importance of sleep. Data were analyzed using Spearman's rho test and Cramer's *V* test. Furthermore, frequency tables and qualitative analyses were employed.

**Results:**

The survey was completed by 427 participants from 34 hospitals in 25 Dutch cities. While healthcare professionals reported sleep to be especially important for neonates admitted in the NICU, low scores were achieved in the area of knowledge of sleep physiology. Most healthcare professionals (91.8%) adapted the timing of elective care procedures to sleep. However, sleep assessments were not based on scientific knowledge. Therefore, the difference between active sleep and wakefulness may often be wrongly assessed. Finally, sleep is rarely discussed between colleagues (27.4% regularly/always) and during rounds (7.5%-14.3% often/always).

**Implications:**

Knowledge about sleep physiology should be increased through education among neonatal healthcare professionals. Furthermore, sleep should be considered more often during rounds and handovers.

With advances in neonatal care, the number of infants surviving preterm birth is progressively increasing.^1^ Brain development is affected by preterm birth,^2^ which can result in future cognitive, motor, and language impairments.^3^ Infant- and family-centered developmental care aims to optimize early brain maturation by integrating different aspects of care that support a healing environment.^4^

The core pillars of infant- and family-centered developmental care include paying close attention to the infant's behavioral cues, parental engagement, and customized adaptations to the NICU (neonatal intensive care unit) and hospital environment.^5^–^9^ Key concepts of developmental care include skin-to-skin contact, supporting the infant during painful procedures, promotion and support of breastfeeding, and protection from deleterious environmental stimuli such as loud noises.^6^,^9^,^10^ This approach has been shown to be beneficial for parental well-being and infant development including brain growth.^7^,^8^ Within this framework, there is a growing recognition of the importance of sleep in the preterm and term born infants for optimal growth and development.^11^,^12^ More specifically, sleep is thought to be the predominant driver of brain activity in the developing fetus^13^,^14^ and preterm infants.

Despite growing awareness of the importance of early life sleep, recognizing the different sleep stages in infants may pose a challenge for healthcare professionals. Neonatal sleep consists of 2 main stages: active sleep and quiet sleep.^15^,^16^ Quiet sleep is easily detected as a sleep stage, since it is characterized by a still posture, closed eyes, and a regular heart and breathing rate.^17^,^18^ Active sleep, however, is characterized by frequent body and facial movements and twitches, rapid eye movements—sometimes with fluttering eyelids—and an irregular heart and breathing rate.^17^,^18^ Because of the appearance of this sleep stage, it can be difficult for the untrained eye to distinguish active sleep from wakefulness.

During active sleep—the neonatal equivalent of REM (rapid eye movement) sleep in older children and adults—endogenous generated brain activity is thought to be crucial for developing brain connectivity.^13^,^14^ In addition, quiet (non-REM) sleep is thought to be related to synaptic downscaling and pruning later in development.^19^ However, no human studies have been published on this topic.

The body of knowledge on the importance of sleep during early life and its relationship to brain development is growing.^11^,^12^ Protecting sleep in infants is considered to be an increasingly important factor in developmental care of (pre-)term infants. However, it is unclear to what extent sleep is currently considered in the day-to-day care at the neonatal ward. To our knowledge, no large-scale studies have been published on the subject of sleep education of neonatal healthcare professionals. Furthermore, since sleep is not a mandatory part of the Dutch healthcare education, the level of education and emphasis placed on sleep are unclear. Therefore, the purpose of the current research was to assess the level of knowledge about sleep among neonatal healthcare professionals. This may serve as a baseline for future intervention studies. The ultimate goal was to improve education about sleep among healthcare professionals. Improved education may lead to increased knowledge about sleep physiology and better sleep practices in the neonatal ward in accordance with the concepts of developmental care.

The aims of the current research were (1) to identify the current state of knowledge about neonatal sleep (including neonatal sleep physiology) amongst Dutch healthcare professionals caring for neonates; (2) to investigate to what extent elective care is adapted to neonatal sleep and to what extent neonatal sleep is discussed in Dutch hospitals; and (3) how theoretical knowledge about neonatal sleep is transferred into daily practice.

To investigate these aims, we conducted an e-survey among Dutch healthcare professionals working with neonates. The current research was designed to adhere to the SQUIRE-guidelines^20^ (see Supplemental Digital Content Appendix A, available at: http://links.lww.com/ANC/A222, for the checklist), as, in the future, we aim to use this work as a quality improvement initiative in neonatal care.

**What This Study Adds**

- Care is often adapted to perceived sleep stages. However, sleep assessments are not based on scientific knowledge. Therefore, the difference between active sleep and wakefulness is often wrongly assessed.
- Sleep is barely discussed during rounds and handovers. Tips are given to incorporate sleep in discussions during rounds and handovers.
- Education on sleep and access to automated sleep monitoring algorithms may improve the adaptation of elective care to sleep in the NICU.

## METHODS

### Participants and Data Acquisition

Between March 2021 and November 2021, a sleep practices and physiology survey was distributed electronically to Dutch healthcare professionals using our NICU nurses' and medical doctors' network via e-mail and via social media. Only healthcare professionals working in Dutch hospitals who are directly involved in neonatal care were invited to participate. Most participants were nurses, neonatologists, and members of the extended multidisciplinary team. Because of the nature of the study, approval by an institutional review board was not required.

### The Survey

The survey used was derived from a study conducted by Hulst et al^21^ investigating the level of knowledge and application of sleep health practices by healthcare professionals working in pediatric rehabilitation. This survey was adapted by experts in neonatal sleep (J.D., A.H.) to suit the population group of the current research. A pilot study (n = 60) was conducted in the Wilhelmina Children's Hospital, Utrecht, to assess feasibility among this specific group of healthcare professionals working with neonates. The survey was adapted afterward. The adapted survey was used for the current study and published as a Google Form.

The survey (see Supplemental Digital Content Appendix B, available at: http://links.lww.com/ANC/A223) consisted of both open and closed questions, with closed questions seeking answers from multiple-choice, yes/no, true/false, and Likert scale questions. The survey was divided into 3 distinct categories. These categories included (1) demographics; (2) practical questions; and (3) objective knowledge questions.

### Data Analysis

Results of the adapted survey were analyzed using SPSS (released 2019; IBM SPSS Statistics for Windows, version 26.0.0.1; IBM Corp, Armonk, New York).

Frequency tables were used to explore demographic information, knowledge level, importance of sleep, and the extent to which sleep is considered in care.

Because of the nonparametric qualities of the data, Spearman's rho test was used to investigate (1) the relation between knowledge and demographic information (age, education, and work experience); (2) the relation between self-reported knowledge, tested knowledge, and timing of elective care; and (3) the relation between the self-rated importance of sleep and discussing sleep during handovers/rounds.

The relationship between healthcare professionals (nurses, neonatologists, pediatricians, resident physicians, physician assistants, and fellows) and knowledge, practices, and discussing sleep during handovers/rounds was investigated using Cramer's *V* test. Finally, a number of open questions were qualitatively analyzed.

## RESULTS

### Demographics

The survey was completed by 432 participants from 34 hospitals in 25 Dutch cities. Of the 34 hospitals, 8 were academic hospitals (level III; n = 114) and 27 were peripheral hospitals (level II; n = 317). Five respondents were eliminated from the study as they either had no experience in neonatal healthcare (n = 3) or did not work in a Dutch hospital (n = 2). Demographics are presented in Table 1.

**TABLE 1.** Group Characteristics

	n	%
Participants	427	
Age		
20-30 y	93	21.8
30-40 y	95	22.2
40-50 y	100	23.4
50-60 y	111	26.0
60+ y	28	6.6
Education		
Secondary school	3	0.7
Secondary vocational education	69	16.2
Higher professional education	210	49.2
University bachelor	10	2.3
University master	135	31.6
Children raised at home		
Yes	300	70.3
Position in hospital		
Resident/physician assistant/fellow	33	7.7
Pediatrician	46	10.8
Neonatologist	50	11.7
Nurse	264	61.8
Other^a^	33	7.8
Missing	1	0.2
Experience in neonatal healthcare		
0-2 y	58	13.6
2-10 y	100	23.4
10-20 y	121	28.3
>20 y	148	34.7
Sleep knowledge acquired at		
Study	23	5.4
Work/practice	261	61.1
Both study and work/practice	129	30.2
Other^b^	14	3.3

^a^Other professions included care assistant (1×), child physiotherapist (1×), child neurologist (2×), lactation nurse (2×), manager (3×), master's student Technical Medicine (1×), maternity nurse (7×), medical pedagogical expert (12×), nurse practitioner (3×), and researcher (1×).

^b^Specified in text.

### Level of Knowledge

To test the knowledge that healthcare professionals have on sleep, multiple objective knowledge questions were asked (Table 2). For the most part, participants with a higher level of education, a higher number of working years, or older in age had higher scores on sleep knowledge. Nevertheless, lower age was related to a higher score on the true/false statement “Adults have relatively less deep sleep (nREM) compared to infants” (rho =−0.108, *P* = .025). Furthermore, less work experience with neonates was related to a higher score on the question, “When are you sure an infant is awake compared to active sleep?” (rho =−0.126, *P* = .009). Finally, physician assistants, resident physicians, and fellows could not distinguish sleep from wakefulness better than neonatologists, nurses, and pediatricians (Cramer's *V* = 0.128, *P* = .051). For a full overview of all associations between demographic characteristics and the objective knowledge questions, see Supplemental Digital Content Table 1 (available at: http://links.lww.com/ANC/A224).

**TABLE 2.** Knowledge of Sleep

	Per 24 h: How Much Does an a-Term Born Infant Sleep Between 0 and 3 mo?	Per 24 h: How Much Does a Baby Born at 30 wk Sleep?	Preterm Infants Sleep More Compared to a Term Infants.^a^	What Does the Sleep Cycle of an Adult Look Like?	True or False: The Sleep Cycle of an Adult Lasts Shorter Compared to an Infant's Sleep Cycle.	True or False: Adults Have Relatively Less Non-REM (Quiet) Sleep Compared to Infants.	What Are Current Insights About Active Sleep?	When Are You Sure an Infant is Awake Compared to Being in Active Sleep? When an Infant...
n	427	427	427	427	427	427	401	427
Missing	0	0	0	0	0	0	26	0
Median	−1	0	1	1	1	0	0	−1
Range	−1 to 1	−1 to 1	−1 to 1	−2 to 2	−1 to 1	−1 to 1	0-2	−2 to 1
% correct no.^b^	42.6%	44.7%	77.3%	1 = 33.5% 2 = 33.0%	55.5%	38.6%	1 = 33.5% 2 = 12.6%	14.3%

Abbreviation: REM, rapid eye movement.

^a^This was not a question but part of the analysis. The answers of the question, “Per 24 hours: How much does a term born infant sleep between 0 and 3 months?” and the question, “Per 24 hours: How much does an infant born at 30 weeks sleep?” were compared to see whether people knew whether preterm infants slept more than term-born infants. If this was the case, the answer was scored as “1.” The sleep time of term-born infants was estimated higher than the sleep time of preterm infants, and the answer was scored as “−1.” If the sleep time of term-born infants and preterm infants was the same, the answer was scored as “0.”

^b^Answers to knowledge questions were scored on a scale, giving each correct answer +1 point and each incorrect answer −1 point. If participants responded they did not know the answer, a score of “0” was given. The sum of all answers is the final score per question. For example, an answer has 4 possibilities: 2 correct and 2 incorrect. A participant gave 1 correct (+1) and 2 incorrect (−2) answers, resulting in a score of −1. If 2 correct answers are possible, the percentage is given for 1 correct answer (+1 score) and for giving both correct answers (+2 score).

**FIGURE 1:**
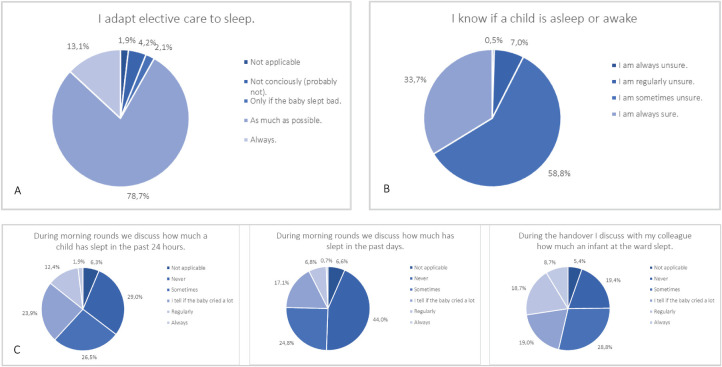
Sleep practices.

Respondents who worked at an academic (level III) hospital were older (rho =−0.16, *P* = 0.001), had higher education levels (rho =−0.15, *P* =.002), and knew more about the importance of active sleep for development (rho =−0.16, *P* = .001). Almost all respondents considered sleep to be important for growth, the immune system, recovery, brain development, and digestion (Table 3). Furthermore, for most healthcare professionals, discussing sleep is not daily practice during rounds (often/always = 7.5%/14.3%), nor during the handover (often/always = 27.4%) (Figure 1). When sleep is considered important, the chance that it is discussed during the handover increases (rho = 0.113-0.162, *P* = .001-.019; Table 4).

**TABLE 3.** Association Between Importance of Sleep and Discussing Sleep

	Growth	Immune System	Recovery	Brain Development	Digestion
**N**					
**Valid**	427	427	427	427	427
**Missing**	0	0	0	0	0
**Median**	5	5	5	5	4
**Range**	0-5	0-5	0-5	0-5	0-5
**Quite important, %**	12.9	23.0	11.2	7.5	30.2
**Very important, %**	82.7	64.4	84.3	88.1	45.7

**TABLE 4.** The Importance of Sleep in Infant Health and Well-being^a^

	Discuss Sleep Over Past 24 h During Rounds	Discuss Sleep Over Past Days During Rounds	Discuss Sleep With Colleague During Handover
Importance of sleep for growth			
Spearman's rho	0.058	0.056	0.113
*P*	.231	.246	.019^**^
Importance of sleep for immune system			
Spearman's rho	0.030	0.066	0.089
*P*	.543	.174	.066
Importance of sleep for recovery			
Spearman's rho	0.102	0.084	0.059
*P*	.035*	.084	.226
Importance of sleep for brain development			
Spearman's rho	0.021	0.020	0.021
*P*	.663	.681	.670
Importance of sleep for digestion			
Spearman's rho	0.140	0.114	0.162
*P*	.004^**^	.018^*^	.001^**^

^a^All significant *P* values are indicated with an asterisk, with one asterisk (*) meaning *P* < .05 and 2 asterisks (**) meaning *P* < .01.

However, sleep is only discussed more frequently during rounds if it is considered to be important for recovery (rho = 0.102, *P* = .035) or digestion (rho = 0.114, *P* = .018 [past days]; rho = 0.140, *P* = .004 [past 24 hours]). There was no relationship between discussing sleep during rounds/handover and whether sleep is considered important for brain development (rho = 0.021, *P* = .663; rho = 0.020, *P* = .681; rho = 0.021, *P* = .670). Finally, nurses discuss sleep during the handover more often than all other healthcare professionals (pediatricians, neonatologists and physician assistants, resident physicians, and fellows) (Cramer's *V* = 0.308, *P* < .000).

### Sleep in Healthcare Practice

To test the way sleep was considered in healthcare practices on the neonatal ward, several additional questions were asked. Most healthcare professionals indicate that they adapt elective care to sleep (eg, delay care when an infant is asleep) either always (13.1%) or as much as possible (78.7%). Hospital level was not associated with adapting sleep to care in practice (rho = 0.07, *P* = .16). However, most healthcare professionals indicate they always (0.5%), often (7.0%), or sometimes (58.8%) doubt whether an infant is awake or asleep. For a complete overview of all questions about sleep practices, see Figure 1.

**FIGURE 2:**
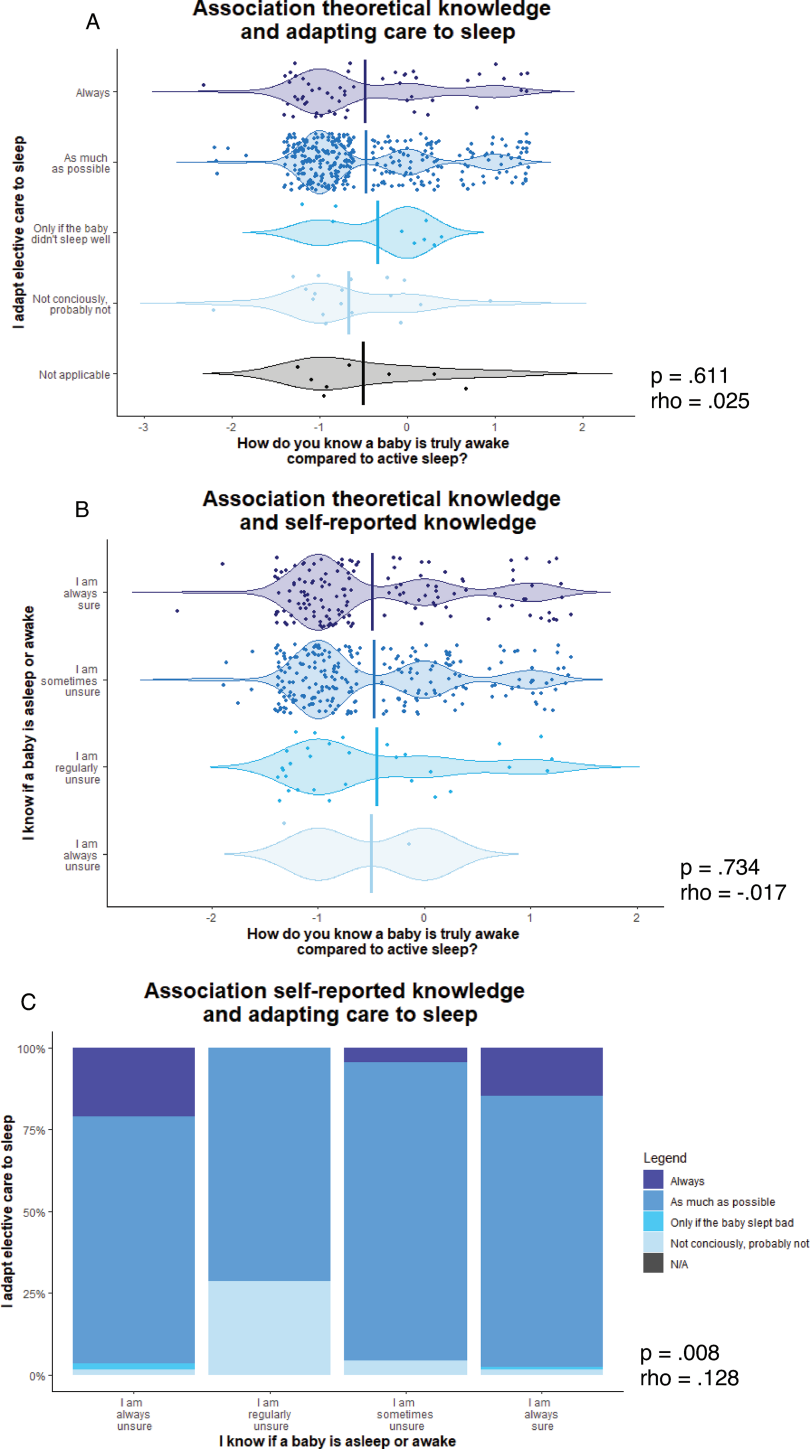
The association between objective knowledge, self-reported knowledge, and adapting elective care to sleep.

Higher self-reported certainty of whether an infant is awake or asleep is associated with more frequent adaptation of elective care to sleep in accordance to developmental care principles (rho = 0.128, *P* = .008). However, both self-reported knowledge whether an infant is asleep (rho =−0.017, *P* = .734) and adapting elective care to sleep (rho = 0.025, *P* = .611) show no association with the knowledge question, “When are you sure an infant is awake compared to active sleep?” (Figure 2). Interestingly, healthcare professionals working at an academic (level III) hospital scored higher on the theoretical knowledge question about recognizing active sleep (rho =−0.11, *P* = 0.03). However, healthcare professionals working at a peripheral (level II) hospital reported more often that they were able to distinguish active sleep from wake (rho = 0.21, *P* < .000).

### Origin of Knowledge

Regarding the origin of knowledge, 61.1% (n = 261) of participants indicated that knowledge about sleep was acquired from the workplace. In 5.4% (n = 23) of respondents, it was indicated that knowledge was acquired from education, while 30.2% (n = 129) indicated their knowledge was acquired from both workplace and education. Findings showed 63 respondents (15.0%) indicated they had acquired knowledge about sleep from other sources, mostly from personal experience as they had children of their own. A total of 22 respondents indicated they had acquired knowledge via scientific literature, conferences, and additional education. In this study, 17 respondents indicated to have read popular literature (n = 8) or articles on the Internet (n = 9), one respondent indicated to be educated by colleagues, and finally one of the respondents replied they relied on intuition.

### General Remarks

The opportunity to give a general remark at the end of the survey was used by 39 of 428 respondents. After analysis of these remarks, it was clear that knowledge regarding neonatal sleep is considered very important and sharing this knowledge—for example, by education, scientific outreach—is key. For example, one respondent wrote: “Something to think about during rounds,” and another respondent wrote, “Specific questions that make you think” (both quotes are translated from Dutch).

## DISCUSSION

This study describes state of knowledge about sleep and the extent to which sleep is considered in Dutch neonatal care by healthcare professionals working with neonates using an online survey. Results show that healthcare professionals consider sleep to be especially important for (pre-)term infants. More specifically, sleep was identified as important for growth, the immune system, recovery, brain development, and digestion. Most healthcare professionals (91.8%) working in neonatal care indicated that they consider sleep in relation to timing of elective care procedures in accordance with developmental care principles, that is, not unnecessarily disturbing the infants during sleep. Nevertheless, healthcare professionals had low scores on objective knowledge questions regarding sleep physiology. Moreover, sleep is barely discussed between colleagues and during daily ward rounds.

Results showed that, using self-assessment, one-third of healthcare professionals were always certain whether an infant was asleep or awake, while half of the respondents sometimes knew whether an infant was asleep or awake. However, less than 15% of all respondents gave a correct answer to the objective knowledge question assessing whether participants knew the difference between wake and active sleep. This corresponds with a similar small study, including only 30 nurses, finding that 20% of nurses were able to select the correct characteristics of active sleep.^22^ Findings indicate that self-reported knowledge may not always align with knowledge tested using science-based questions, with participants tending to “overclaim” their personal expertise favorably, in other words, “overprecision.”^23^ In the context of neonatal sleep, it is difficult to correctly distinguish wake and sleep, since active sleep may resemble waking activity to inexperienced observers.^17^,^18^

Higher self-reported knowledge was related to more frequent adaption of elective care to sleep. This might imply that healthcare professionals may adapt elective care to their own perception of sleep and wake. However, neither self-reported knowledge nor adaption of elective care was associated with giving the right or wrong answer to the objective knowledge question, “When are you sure an infant is awake compared to being in active sleep?” Therefore, the infant may still be unnecessarily disturbed and woken up for care procedures due to a lack of expertise and knowledge on the difference between being awake and being in active sleep. This specific phenomenon has, to our knowledge, not previously been studied in neonates. Previous work complements this finding, describing a decrease in the quality of sleep in hospital among children between 1 and 18 years old^24^ and adults^25^ is largely attributed to awakenings by staff for care procedures.

Participants reported to have acquired their sleep knowledge via multiple sources, with work experience and education being prefilled answers. From these two, work experience was most commonly reported to be the source of knowledge about sleep. The work/education dichotomy, knowledge on neonatal sleep was mainly acquired via one's private life, for example, when raising children of their own. Interestingly, less work experience is associated with higher scores on objective knowledge questions. Besides, physician assistants, resident physicians, and fellows were better able to distinguish sleep from wake than neonatologists, nurses, and pediatricians. The former group consists of healthcare professionals who are at the start of their careers; thus, they might possess more knowledge from recent education. Given the increased interest in neonatal and preterm sleep,^11^,^12^ recent education might include more information about this subject. Acquiring knowledge at the workplace poses risks including healthcare professionals acting on insufficient or outdated knowledge. Furthermore, this poses the need for education—both healthcare curricula and refresher training—to teach new scientific findings regarding sleep.

Nevertheless, knowledge acquisition in the workplace should not be discarded. Bedside teaching is shown to have several benefits, including increased active learning, motivation, patients' understandings, problem-solving, and decision-making skills.^26^ If healthcare professionals know more about sleep, they can teach parents how to support infant sleep both in the neonatal unit and at home. However, in the study of Hulst et al,^21^ parents were found to have a similar level of knowledge about sleep compared with healthcare professionals, again stressing the need for educating healthcare professionals on sleep.

Sleep is infrequently discussed during rounds despite developmental care practices being common in Dutch healthcare settings. Infant- and family-centered developmental care is focused on supporting sleep by timing procedures around the sleep cycle, involving parents in settling the infant to sleep, creating a quiet, calm, and dark environment, and encouraging skin-to-skin care, which may improve sleep organization.^10^ To optimally apply these strategies, there is a need for greater discussion of sleep among the neonatal clinical team and parents.

### Strengths and Limitations

A limitation is that the survey used was not entirely validated. To ensure the reliability and replicability, several actions were undertaken. The survey was first formulated on the basis of a survey developed by Hulst et al.^21^ After receiving feedback on this survey from our pilot study, changes were made accordingly. Furthermore, when analyzing the results, the team always worked in pairs of 2 or 3 during the qualitative analyses to avoid any bias.

To the best of our knowledge, this research stands out as the first nationwide study to evaluate sleep practices and knowledge among neonatal healthcare professionals. One of its key strengths lies in this aspect. The survey is filled in by a large number and a high variety of healthcare professionals throughout the country, including both academic and peripheral hospitals. Furthermore, the survey itself clearly contributed to the distribution of knowledge and served as a reminder to the importance of sleep. For example, one respondent wrote: “Something to think about during rounds,” and another respondent wrote, “Specific questions that make you think” (both quotes are translated from Dutch). With this, the survey already contributed to our final goal of increasing awareness and knowledge about neonatal sleep in hospitals.

### Future Research

Currently (pre-)term infant sleep—and therefore developmental care—may be disturbed because of lack of knowledge about sleep and sleep behavior in the neonatology ward. More knowledge about neonatal sleep may cause a plethora of positive changes. When healthcare professionals are knowledgeable on infant sleep physiology, they are more likely to educate parents in this area. This way, parent-based developmental care can continue beyond the hospital into the infant's home. Important sources of knowledge may be behavioral sleep scales^17^,^18^ and the NIDCAP (Newborn Individualized Developmental Care and Assessment Program) or FINE (Family and Infant Neurodevelopment Education) manuals.^6^,^9^,^10^ Future research should focus on the best way for healthcare professionals and parents to acquire this knowledge.

A small step in this direction has already been made by our group. A Dutch e-Learning course about pediatric sleep is available and is promoted as refresher training for professionals and in general for nonprofessionals. This may be beneficial to all healthcare professionals, as it is shown that sleep knowledge can be successfully increased through provision of sleep education, both when delivered face-to-face and through online webinars.^27^ In addition, Toivonen et al^28^ found that the quality of family-centered care was successfully improved in multiple hospitals by an educational intervention that utilizes a mentor system. Based on their findings, a one-on-one personal mentor program could further increase sleep-protective care.

Preterm infants have a higher rate of sleep difficulties than term born infants, as was extensively reviewed by Bennet et al.^29^ However, to the best of our knowledge, no evidence-based guidelines have been published specifically for infants during the period after the hospital (NICU) stay. Even for healthy infants, currently little evidence exists regarding interventions to promote good sleep.^30^,^31^ Furthermore, most interventions developed to improve infant sleep in the first 6 months after birth seem to mainly target parent mood instead of infant sleep quality.^32^ Future research should aim to develop evidence-based practice guidelines for infant sleep and more specifically for infant sleep after NICU discharge.

### Recommendations for Practice

Two areas for improvement were identified. First, knowledge on sleep physiology should be improved. Most healthcare professionals can accurately describe the importance of sleep and identify areas for which sleep may be important. However, to adapt elective care to sleep, healthcare professionals should also be able to assess sleep stages based on the current scientific consensus.

Recently, our research group published a review^18^ and a behavioral scoring system^17^ on sleep stages. It should be noted that the current consensus about the appearance of active sleep may resemble discomfort, according to frequently used comfort scales.^33^ However, discussions on our ward have shown that, with close examination, healthcare professionals are able to distinguish active sleep in infants who show signs of discomfort. In healthcare curricula, a module on sleep can be added.

Besides individual study of scientific sources on sleep, both formal education and refresher training should include a module about sleep. This is currently been implemented in a refresher training for nurses in our hospital and yields very positive reactions and more awareness about sleep during care. The refresher training comprises a 1-hour lecture about the importance of sleep, the biological background of sleep, the development of sleep and sleep stages, and the appearance of sleep stages in preterm infants.

Second, sleep is infrequently discussed during rounds and handovers. To increase discussions about sleep between colleagues, it is important to have insight into the sleep schedule of infants in the NICU. It is not possible to continuously manually assess sleep. Currently, several algorithms exist to assess preterm sleep stages.^34^ However, these still have to be validated on the bedside.

Nevertheless, sleep can still be discussed during handovers without continuous monitoring to increase awareness and understanding of sleep. If the ABCDE method (structured assessment of Airway, Breathing, Circulation, Disability, Exposure) is used, sleep can be discussed during Disability, when neurology is discussed, or during Exposure, when environment is discussed. It depends on the hospital and the practitioner when things such as pain and comfort are discussed. Of course, it is important to stay objective in your assessment. For example, we recommend to not say an infant was “sound asleep” but to describe which behavior you saw during checks (eg, lots of movements or not at all), how easily the infant woke up during care procedures, and/or whether the infant was groggy or alert. Finally, we recommend to discuss sleep at the end of the discussion, so all things that may have disturbed sleep (eg, medical procedures, bradycardias, or apneas) are known.

## CONCLUSION

Healthcare professionals are highly motivated and willing to adapt elective care to optimize sleep in neonates admitted to the neonatology department. However, these current practices do not seem to be based on theoretical background. General scores on theoretical knowledge questions were low. Besides, healthcare professionals who scored high on theoretical knowledge questions were not more likely to adapt elective care to optimize sleep. Therefore, knowledge about neonatal sleep among healthcare professionals should be improved using education in curricula and refresher training.

**Table:** Summary of Recommendations for Practice and Research

**What we know:**	Sleep is an important behavioral stage that is hypothesized to facilitate and guide development. Sleep is considered especially important for neonates admitted to the NICU.
**What needs to be studied:**	A way to continuously monitor sleep in infants admitted to the hospital. The direct effect of sleep education and increased awareness on sleep quality of infants admitted to the hospital.
**What can we do today:**	Sleep should be discussed more frequently during rounds and handovers. Sleep education should get more attention both during formal education and during refresher training. It is important to transfer this knowledge to parents and include them in sleep-protective care.

## Supplementary Material

SUPPLEMENTARY MATERIAL

## References

[1] Costeloe KL Hennessy EM Haider S Stacey F Marlow N Draper ES. Short term outcomes after extreme preterm birth in England: comparison of two birth cohorts in 1995 and 2006 (the EPICure studies). BMJ. 2012;345:e7976. doi:10.1136/bmj.e7976. 23212881 10.1136/bmj.e7976 PMC3514472

[2] Bouyssi-Kobar M du Plessis AJ McCarter R Third trimester brain growth in preterm infants compared with in utero healthy fetuses. Obstet Gynecol Surv. 2017;72(3). https://journals.lww.com/obgynsurvey/Fulltext/2017/03000/Third_Trimester_Brain_Growth_in_Preterm_Infants.3.aspx. Accessed June 7, 2023. 10.1542/peds.2016-1640 PMC5079081 27940782

[3] Stipdonk LW Boumeester M Pieterman KJ Cerebellar volumes and language functions in school-aged children born very preterm. Pediatr Res. 2021;90(4):853–860. doi:10.1038/s41390-020-01327-z. 33469182 10.1038/s41390-020-01327-z

[4] European standards of care for newborn health. Infant- & family-centered developmental care: overview. https://www.efcni.org/activities/projects/escnh. Published 2021. Accessed June 7, 2023.

[5] Als H. A synactive model of neonatal behavioral organization. Phys Occup Ther Pediatr. 1986;6(3/4):3–53. doi:10.1080/J006v06n03_02.

[6] NIDCAP. Program guide—Newborn Individualized Developmental Care and Assessment Program (NIDCAP). An education and training program for health care professionals. https://nidcap.org/wp-content/uploads/2021/03/Program-Guide-Rev-30Mar2021.pdf. Published 2021. Accessed June 7, 2023.

[7] Als H Duffy FH McAnulty G NIDCAP improves brain function and structure in preterm infants with severe intrauterine growth restriction. J Perinatol. 2012;32(10):797–803. doi:10.1038/jp.2011.201. 22301525 10.1038/jp.2011.201 PMC3461405

[8] Westrup B. Family-centered developmentally supportive care. Neoreviews. 2014;15(8):e325–e335. doi:10.1542/neo.15-8-e325.

[9] Smith K Buehler D Als H. Nursery Assessment Manual. NIDCAP Nursery Program. Woburn, MA: NIDCAP Federation International (NFI); 2019. https://nidcap.org/wp-content/uploads/2019/01/NIDCAP-Nursery-Program-Guide-Rev-31Jan2019.pdf. Accessed June 7, 2023.

[10] Warren I. Foundation Toolkit of Family Centered Developmental Care. St Albans, Herts, England: FINE Partnership; 2015.

[11] Mason GM Lokhandwala S Riggins T Spencer RMC. Sleep and human cognitive development. Sleep Med Rev. 2021;57:101472. doi:10.1016/j.smrv.2021.101472. 33827030 10.1016/j.smrv.2021.101472 PMC8164994

[12] Trickett J Hill C Austin T Johnson S. The impact of preterm birth on sleep through infancy, childhood and adolescence and its implications. Children (Basel). 2022;9(5):626. doi:10.3390/children9050626. 35626803 10.3390/children9050626 PMC9139673

[13] Blumberg MS Dooley JC Sokoloff G. The developing brain revealed during sleep. Curr Opin Physiol. 2020;15:14–22. doi:10.1016/j.cophys.2019.11.002. 32864534 10.1016/j.cophys.2019.11.002 PMC7450535

[14] Del Rio-Bermudez C Blumberg MS. Active sleep promotes functional connectivity in developing sensorimotor networks. Bioessays. 2018;40(4):e1700234. doi:10.1002/bies.201700234. 29508913 10.1002/bies.201700234 PMC6247910

[15] Mirmiran M Maas YGH Ariagno RL. Development of fetal and neonatal sleep and circadian rhythms. Sleep Med Rev. 2003;7(4):321–334. doi:10.1053/smrv.2002.0243. 14505599 10.1053/smrv.2002.0243

[16] Peirano P Algarín C Uauy R. Sleep-wake states and their regulatory mechanisms throughout early human development. J Pediatr. 2003;143(4)(suppl):S70–S79. doi:10.1067/s0022-3476(03)00404-9. 14597916 10.1067/s0022-3476(03)00404-9

[17] de Groot ER Bik A Sam C Creating an optimal observational sleep stage classification system for very and extremely preterm infants. Sleep Med. 2022;90:167–175. doi:10.1016/j.sleep.2022.01.020. 35182976 10.1016/j.sleep.2022.01.020

[18] Bik A Sam C de Groot ER A scoping review of behavioral sleep stage classification methods for preterm infants. Sleep Med. 2022;90:74–82. doi:10.1016/j.sleep.2022.01.006. 35123149 10.1016/j.sleep.2022.01.006

[19] Knoop MS de Groot ER Dudink J. Current ideas about the roles of rapid eye movement and non-rapid eye movement sleep in brain development. Acta Paediatr. 2021;110(1):36–44. doi:10.1111/apa.15485. 10.1111/apa.15485 PMC7818400 32673435

[20] Goodman D Ogrinc G Davies L Explanation and elaboration of the SQUIRE (Standards for Quality Improvement Reporting Excellence) Guidelines, V.2.0: examples of SQUIRE elements in the healthcare improvement literature. BMJ Qual Saf. 2016;25(12):e7. doi:10.1136/bmjqs-2015-004480. 10.1136/bmjqs-2015-004480 PMC5256235 27076505

[21] Hulst RY Pillen S Voorman JM Rave N Visser-Meily JMA Verschuren O. Sleep health practices and sleep knowledge among healthcare professionals in Dutch paediatric rehabilitation. Child Care Health Dev. 2020;46(6):703–710. doi:10.1111/cch.12799. 32706911 10.1111/cch.12799 PMC7589250

[22] Mahmoodi N Arbabisarjou A Rezaeipoor M Pishkar Mofrad Z. Nurses' awareness of preterm neonates' sleep in the NICU. Glob J Health Sci. 2015;8(6):226–233. doi:10.5539/gjhs.v8n6p226. 26755487 10.5539/gjhs.v8n6p226 PMC4954870

[23] Moore DA. Overprecision is a property of thinking systems [published online ahead of print, May 5, 2022]. Psychol Rev. doi:10.1037/rev0000370. 10.1037/rev0000370 35511530

[24] Hybschmann J Topperzer MK Gjærde LK Sleep in hospitalized children and adolescents: a scoping review. Sleep Med Rev. 2021;59:101496. doi:10.1016/j.smrv.2021.101496. 33984632 10.1016/j.smrv.2021.101496

[25] Growdon ME Inouye SK. Minimizing sleep disruption for hospitalized patients: a wake-up call. JAMA Intern Med. 2018;178(9):1208–1209. doi:10.1001/jamainternmed.2018.2679. 30014149 10.1001/jamainternmed.2018.2679

[26] Salam A Siraj HH Mohamad N Das S Rabeya Y. Bedside teaching in undergraduate medical education: issues, strategies, and new models for better preparation of new generation doctors. Iran J Med Sci. 2011;36(1):1–6. https://pubmed.ncbi.nlm.nih.gov/23365470. Accessed June 7, 2023. 23365470 PMC3559110

[27] Osborne JM Blunden S. Evaluating accessible sleep health information in rural and urban contexts: delivery face-to-face or online? Clin Med Insights Pediatr. 2018;12:1179556518815168. doi:10.1177/1179556518815168. 30574004 10.1177/1179556518815168 PMC6295703

[28] Toivonen M Lehtonen L Löyttyniemi E Ahlqvist-Björkroth S Axelin A. Close Collaboration with Parents intervention improves family-centered care in different neonatal unit contexts: a pre–post study. Pediatr Res. 2020;88(3):421–428. doi:10.1038/s41390-020-0934-2. 32380505 10.1038/s41390-020-0934-2 PMC7478938

[29] Bennet L Walker DW Horne RSC. Waking up too early—the consequences of preterm birth on sleep development. J Physiol. 2018;596(23):5687–5708. doi:10.1113/JP274950. 29691876 10.1113/JP274950 PMC6265542

[30] Field T. Infant sleep problems and interventions: a review. Infant Behav Dev. 2017;47:40–53. doi:10.1016/j.infbeh.2017.02.002. 28334578 10.1016/j.infbeh.2017.02.002

[31] Douglas PS Hill PS. Behavioral sleep interventions in the first six months of life do not improve outcomes for mothers or infants: a systematic review. J Dev Behav Pediatr. 2013;34(7):497–507. doi:10.1097/DBP.0b013e31829cafa6. 24042081 10.1097/DBP.0b013e31829cafa6

[32] Kempler L Sharpe L Miller CB Bartlett DJ. Do psychosocial sleep interventions improve infant sleep or maternal mood in the postnatal period? A systematic review and meta-analysis of randomised controlled trials. Sleep Med Rev. 2016;29:15–22. doi:10.1016/j.smrv.2015.08.002. 26555938 10.1016/j.smrv.2015.08.002

[33] Meesters NJ Dilles T van Rosmalen J van den Bosch GE Simons SHP van Dijk M. COMFORTneo scale: a reliable and valid instrument to measure prolonged pain in neonates? J Perinatol. 2023;43(5):595–600. doi:10.1038/s41372-023-01628-1. 36746985 10.1038/s41372-023-01628-1

[34] Sentner T Wang X de Groot ER The Sleep Well Baby project: an automated real-time sleep–wake state prediction algorithm in preterm infants. Sleep. 2022;45(10):zsac143. doi:10.1093/sleep/zsac143. 35749799 10.1093/sleep/zsac143 PMC9548667

