# Supplementary material for: Evaluation of Sleep Practices and Knowledge in Neonatal Healthcare
Source: Adv Neonatal Care. 2023 Aug 14;23(6):499–508. doi: 10.1097/ANC.0000000000001102 (PMC10686278; doi:10.1097/ANC.0000000000001102)
Supplement: SUPPLEMENTARY MATERIAL [file ancr-23-0499-s002.docx]

# Supplement B – The survey

*Link to original (Dutch) survey:* [*https://forms.gle/37kJmUm5pbThRFQq8*](https://forms.gle/37kJmUm5pbThRFQq8)

**How much do you know about sleep?**

Adults sleep most of the night. A baby that has to grow and develop add quite a few hours more. I won’t give too much away about the importance of sleep, since I want to ask you to fill in the following survey about your knowledge on sleep.

Aim: The aim of this survey is assessing the amount of knowledge of the average Dutch health care professional, working with babies. Therefore we ask you NOT to guess answers if you are not sure. In that case you can fill in the “I don’t know” option.

Anonymous: The survey is anonymous. Therefore your name won’t be asked for this research. After analysis, the results will be published.

Correct answers: At the end of the survey you get the choice to fill in you e-mail address to get more information about a lecture from sleep expert dr. Jeroen Dudink from the University Medical Centre Utrecht. Your e-mail address won’t be processed with the answers and will only be used to share information with you. If you prefer not to share your email address in this survey, you can contact us via the email address that will be shared with you at the end of the survey.

Time: About 7 minutes

Thank you very much for participating!

**About you**

*What is your age?*

1. 15-20 years
2. 20-30 years
3. 30-40 years
4. 40-50 years
5. 50-60 years
6. 60+ years

*What is you highest finished education?*

1. Elementary school
2. Secondary school
3. Secondary vocational education (MBO)
4. Higher professional education (HBO)
5. University bachelor
6. University master

*Did you raise children at home from birth?*

1. Yes
2. No

*Do you work in the hospital?*

1. Yes
2. No
3. Yes, for a temporary internship

**Work outside the hospital**

*Do you work with babies? If yes, what kind?*

1. No, not at all
2. Daycare
3. Other, namely … (fill in)

**Work inside the hospital**

*In which hospital do you work?*

1. UMCU/WKZ
2. Diakonessenhuis
3. St. Antonius Ziekenhuis Nieuwegein
4. Other, namely … (fill in)

*In which city?*

1. Utrecht
2. Other, namely … (fill in)

*What is your job in the hospital?*

1. Nurse
2. Neonatologist
3. Pediatrician
4. Other, namely … (fill in)

*How much work experience do you have with babies?*

1. No work experience
2. 0-2 years
3. 2-10 years
4. 10-20 years
5. Over 20 years

**Sleep knowledge**

*Per 24 hours: How much does an a-term born infant sleep between 0 and 3 months?*

1. 14-16 hours
2. 16-18 hours
3. 18-20 hours
4. 20-22 hours
5. 22-24 hours
6. I don’t know (please don’t guess)

*Per 24 hours: How much does a baby born at 30 weeks sleep?*

1. 14-16 hours
2. 16-18 hours
3. 18-20 hours
4. 20-22 hours
5. 22-24 hours
6. I don’t know (please don’t guess)

*What does the sleep cycle of an adult look like? (Multiple answers possible)*

1. 60 minutes
2. 90 minutes
3. More REM than non-REM sleep
4. More non-REM than REM sleep
5. I don’t know (please don’t guess)

*True or false: The sleep cycle of an adult lasts shorter compared to an baby’s sleep cycle.*

1. True
2. False
3. I don’t know (please don’t guess)

*True or false: Adults have relatively less non-REM (quiet) sleep compared to babies.*

1. True
2. False
3. I don’t know (please don’t guess)

*What are current insights about active sleep? (Multiple answers possible)*

1. Active sleep facilitates brain development by brain stimulation
2. Active sleep plays an important role in remembering movements
3. I don’t know (please don’t guess)

*When are you sure an infant is awake compared to being in active sleep? When an infant...*

1. Cries
2. Has open eyes
3. Moves a lot
4. All of the above
5. None of the above
6. I don’t know (please don’t guess)

*How important is the role of sleep (for infants) in the following subjects?*

|  | Not important | A little important | Neutral | Quite important | Very important | I don’t know |
| --- | --- | --- | --- | --- | --- | --- |
| Growth |  |  |  |  |  |  |
| Immune system |  |  |  |  |  |  |
| Recovery |  |  |  |  |  |  |
| Brain development |  |  |  |  |  |  |
| Digestion |  |  |  |  |  |  |

**Experience at the ward**

Please answer as honestly as possible, that way we can learn if there is room for improvement and which improvements would be best for the babies.

*I adapt elective care to sleep.*

1. Never, that is impossible
2. Not consciously (probably not)
3. Only if the baby slept bad
4. Not consciously (probably I do)
5. As much as possible
6. Always
7. Not applicable

*During morning rounds we discuss how much a child has slept in the past 24 hours*

1. Never
2. Sometimes
3. I tell if the baby cried a lot
4. Regularly
5. Always
6. Not applicable

*During morning rounds we discuss how much a child has slept in the past days*

1. Never
2. Sometimes
3. I tell if the baby cried a lot
4. Regularly
5. Always
6. Not applicable

*During the handover I discuss with my colleague how much an infant at the ward slept*

1. Never
2. Sometimes
3. I tell if the baby cried a lot
4. Regularly
5. Always
6. Not applicable

*What is important for the development of a baby on the ward?*

|  | Not important | A little important | Neutral | Quite important | Very important | I don’t know |
| --- | --- | --- | --- | --- | --- | --- |
| Nutrition |  |  |  |  |  |  |
| Rest |  |  |  |  |  |  |
| Sleep |  |  |  |  |  |  |
| Stimulation |  |  |  |  |  |  |
| Clear day/night rhythm |  |  |  |  |  |  |
| Touch from parents |  |  |  |  |  |  |
| Kangaroo care |  |  |  |  |  |  |

*I know if a child is asleep or awake.*

1. I am always unsure
2. I am regularly unsure
3. I am sometimes unsure
4. I am always sure

**Thank you for completing the survey!**

*Where did you mainly acquire your sleep knowledge? (Multiple answers possible)*

1. During my study
2. At the workplace
3. Other, …

*Would you like to learn more about sleep?*

(1) No, thank you

2

3

4

(5) Very much
