# Supplementary material for: Evaluation of Sleep Practices and Knowledge in Neonatal Healthcare
Source: Adv Neonatal Care. 2023 Aug 14;23(6):499–508. doi: 10.1097/ANC.0000000000001102 (PMC10686278; doi:10.1097/ANC.0000000000001102)
Supplement: SUPPLEMENTARY MATERIAL [file ancr-23-0499-s003.docx]

# Supplemental table 1 – The association between knowledge questions and demographics

**Table S1. The association between objective knowledge questions and demographics.** The table shows a significant relation between more knowledge about sleep time and higher age/more work experience. Furthermore, a significant relation was found between more knowledge about the adult sleep cycle and higher education level. More knowledge about the difference between infant and adult sleep was related to a lower age. Finally, more work experience is related to more knowledge about current insights about active sleep (i.e. the role of active sleep), but less knowledge about the difference between active sleep and wake.

|  |  | Age | Highest finished education | Current job in the hospital | Work experience with neonates |
| --- | --- | --- | --- | --- | --- |
| Per 24 hours: How much does an a-term born infant sleep between 0 and 3 months? | Spearman’s rho | -.040 | .020 | .036 | -.051 |
|  | P-value | .405 | .683 | .452 | .290 |
| Per 24 hours: How much does a baby born at 30 weeks sleep? | Spearman’s rho | -.048 | .058 | -.044 | -.017 |
|  | P-value | .320 | .234 | .363 | .731 |
| Preterm infants sleep more compared to a-term infants.† | Spearman’s rho | .107 | -.072 | .027 | .148 |
|  | P-value | .027^*^ | .140 | .575 | .002^**^ |
| What does the sleep cycle of an adult look like? | Spearman’s rho | -.018 | .210 | -.130 | -.045 |
|  | P-value | .705 | .000^**^ | .007^**^ | .356 |
| True or false: The sleep cycle of an adult lasts shorter compared to an baby’s sleep cycle. | Spearman’s rho | -.086 | -.015 | .094 | -.049 |
|  | P-value | .077 | .759 | .052 | .309 |
| True or false: Adults have relatively less non-REM (quiet) sleep compared to babies. | Spearman’s rho | -.108 | .023 | .021 | -.044 |
|  | P-value | .025^*^ | .635 | .671 | .368 |
| What are current insights about active sleep? | Spearman’s rho | .084 | .044 | -.043 | .115 |
|  | P-value | .083 | .360 | .491 | .018^*^ |
| When are you sure an infant is awake compared to being in active sleep? When an infant.... | Spearman’s rho | -.087 | .056 | -.048 | -.126 |
|  | P-value | .073 | .222 | .319 | .009^**^ |

All significant p-values are indicated with an asterisk, with one asterisk (*) meaning p < .05 and two asterisks (**) meaning p < .010.

† This was not a question but part of the analysis. The answers of the question “Per 24 hours: How much does an a-term born infant sleep between 0 and 3 months?” and the question “Per 24 hours: How much does a baby born at 30 weeks sleep?” were compared to see if people knew if preterm infants slept more than term-born infants. If this was the case, the answer was scored as “1”. The sleep time of term-born infants was estimated higher than the sleep time of preterm infants the answer was scored as “-1”. If the sleep time of term-born infants and preterm infants was the same, answer was scored as “0”.
